# Effects of Haloperidol, Risperidone, and Aripiprazole on the Immunometabolic Properties of BV-2 Microglial Cells

**DOI:** 10.3390/ijms22094399

**Published:** 2021-04-22

**Authors:** Valentino Racki, Marina Marcelic, Igor Stimac, Daniela Petric, Natalia Kucic

**Affiliations:** 1 Department of Neurology, Medical Faculty, University of Rijeka, 51000 Rijeka, Croatia; 2 Department of Physiology and Immunology, Medical Faculty, University of Rijeka, 51000 Rijeka, Croatia; mmarcelic@uniri.hr (M.M.); igor.stimac@medri.uniri.hr (I.S.); natalia.kucic@uniri.hr (N.K.); 3 Department of Psychiatry, Medical Faculty, University of Rijeka, 51000 Rijeka, Croatia; daniela.petric@uniri.hr

**Keywords:** antipsychotics, microglia, aripiprazole, risperidone, haloperidol, immunometabolism

## Abstract

Microglial cells are resident macrophages in the brain that have been implicated in the pathophysiology of schizophrenia. There is a lack of studies covering the effects of antipsychotics on microglial cells. The current literature points to a possible anti-inflammatory action without clear mechanisms of action. The aim of this study is to characterize the effects of haloperidol, risperidone and aripiprazole on BV-2 microglial cells in in vitro conditions. We have used immunofluorescence and flow cytometry to analyze the classical pro and anti-inflammatory markers, while a real-time metabolic assay (Seahorse) was used to assess metabolic function. We analyzed the expression of p70S6K to evaluate the mTOR pathway activity with Western blot. In this study, we demonstrate the varying effects of haloperidol, risperidone and aripiprazole administration in BV-2 microglial cells. All three tested antipsychotics were successful in reducing the pro-inflammatory action of microglial cells, although only aripiprazole increased the expression of anti-inflammatory markers. Most significant differences in the possible mechanisms of action were seen in the real-time metabolic assays and in the mTORC1 signaling pathway activity, with aripiprazole being the only antipsychotic to reduce the mTORC1 activity. Our results shed some new light on the effects of haloperidol, risperidone and aripiprazole action in microglial cells, and reveal a novel possible mechanism of action for aripiprazole.

## 1. Introduction

Microglia are cells of the innate immune system that act as resident macrophages in the brain [1]. They originate from the yolk sac in early development and are the first mature cells in the central nervous system [2]. Research into microglial function has been steadily increasing over the last decade, with a wide spectrum of functions discovered and ascribed to them. Available evidence so far points to a clear role in regulating neuronal homeostasis, synaptic pruning, neurogenesis, and the clearance of apoptotic cells [3]. These functions are achieved by the complex interaction of brain cells using various signaling molecules such as cytokines, chemokines, and trophic factors, as well as direct contact of microglia and surrounding cells [4]. Interestingly, the production of neurosteroids by microglial cells was recently characterized, which could be an interesting therapeutic avenue in schizophrenia [5,6]. Microglial function was typically split into resting and active phenotypes, although it is now clear that microglia are consistently active and surveilling the surrounding tissue, even in the considered “resting” state [7]. The two main morphologies of microglia are ramified and ameboid, first signifying the surveilling state, with the latter signifying a proactive state [8]. Microglial morphology roughly matches their function, although this is not always the case, with key differences depending on the position in the brain, as well as the type of stimuli [9]. Therefore, the traditional way of assessing microglial function is becoming outdated, as the whole cellular function integrates both immune and metabolic cell characteristics [10]. Microglia have been implicated in numerous neurodegenerative diseases due to changes in their immunometabolic characteristics and functions [11,12]. Mutations in the key metabolic microglial genes such as myeloid cells-2 gene (Trem2) and granulin gene (Grn) have been identified as risk factors for various neurodegenerative and neuroinflammatory disorders such as Alzheimer’s disease, amyotrophic lateral sclerosis and viral infections of the central nervous system [13]. Microglial cells are at the center of the immune hypothesis of schizophrenia, with the current evidence showing that dysfunctional immune responses lead to altered neuroplasticity and abnormal brain aging [14]. On the other hand, there is a significant amount of research that points in another direction, mostly in translocator protein-18 kDa (TSPO) positron emission tomography–computerized tomography (PET-CT) imaging studies that have contradicting results so far regarding microglial activation in patients suffering from schizophrenia [15].

Schizophrenia is a disorder with an unclear, most likely multifactorial etiology and results from a complex interplay between both genetic and environmental factors [16]. The molecular mechanisms of schizophrenia are not yet elucidated, although it is thought that there is a general hyperexcitation of the central nervous system, mediated by dysregulation in neurotransmitters dopamine and glutamate. In essence, there is a permanent cytotoxic brain state that is caused by excessive oxidative damage and metabolic dysfunction [17]. Antipsychotics are the cornerstone of treatment and are usually split into typical and atypical antipsychotics due to the differences in key mechanisms of action and side-effects. The typical antipsychotics, such as haloperidol, have a firm antagonism of dopamine D_2_ receptors, while the atypical antipsychotics, such as risperidone, have weaker affinity and shorter antagonism of D_2_ receptors. However, the advancement in this field has shown that serotonine 5-HT_2A_ and 5-HT_2C_ receptors are also key targets for antipsychotics, along with muscarinic receptors, H_1_ serotonin receptor antagonism, D_3_ dopamine receptor antagonism, among others [18]. Some antipsychotics, such as aripiprazole, can be considered as third generation antipsychotics as they act in an entirely different manner via partial D_2_ receptor agonism [19]. Regardless of the nomenclature used, each drug has individual characteristics that do not always fit with the mentioned groups.

Most research into the mechanisms of antipsychotics is focused on neurons, with only sparse reports of microglial research. Importantly, microglia possess receptors that are the classical targets for antipsychotics, such as D_2,_ glutamate or 5-HT_2A_ receptors [20]. For example, it is known that metabotropic glutamate receptors (mGluRs) have a varied function depending on the group, where group 2 mGluRs mediate neurotoxic and group 3 mGluRs mediate neuroprotective actions of microglia [21,22]. Likewise, stimulating microglia with serotonin causes a secretion of exosomes, a reaction that could have an impact on functional signaling between neurons and microglia [23]. These two examples prove that neurotransmitters can have a profound impact on microglia, and that any drug that impacts these systems does so in a complex manner affecting both neurons and glial cells.

The available studies of antipsychotic effects on microglia point to a general anti-inflammatory shift upon stimulation. The first published studies by Kowalski and Labuzek focused on the effects of flupentixol, trifluperidol, chlorpromazine and loxapine, where treating activated microglia led to reduced tumor necrosis factor-α (TNF-α), nitrous oxide (NO), interleukin-1β (IL-1β) and interleukin-2 (IL-2) release in a primary microglia culture [24,25]. In a study by Hou et al., olanzapine also inhibited the release of NO from activated microglia, whereas haloperidol and clozapine did not in the N9 microglial cell line [26]. Haloperidol, risperidone, perospirone, ziprasidone and quetiapine inhibited interferon-γ (IFN-γ)-induced microglial activation through reducing inducible NO synthase (iNOS) and TNF-α expression in an in vitro study on a murine 6-3 microglial cell line [27,28]. The same inhibition was seen with aripiprazole stimulation, presumably due to regulating intracellular Ca^2+^ levels via the transient receptor potential melastatin 7 (TRPM7) channels [29,30]. Although the overall number of studies is small, all the mentioned work so far indicates the reduced immune activity of microglia after antipsychotic stimulation. The aim of our research was to further characterize and compare the immunometabolic effects of haloperidol, risperidone and aripiprazole stimulation on BV-2 microglial cells in an in vitro environment.

## 2. Results

Our research was performed on the immortalized murine BV-2 microglial cell line, which was proven to be a suitable alternative model to primary microglia [31], with some caveats regarding differences in the transcriptome and anti-inflammatory signaling properties when compared to primary microglia [32]. We have characterized their properties depending on the cultivation conditions in a previous study, and determined that the cultivation conditions used for this research (DMEM with 10% fetal calf serum supplementations) yield metabolically active ameboid microglia [33].

### 2.1. Cell Viability and Morphology

**Figure 1:**
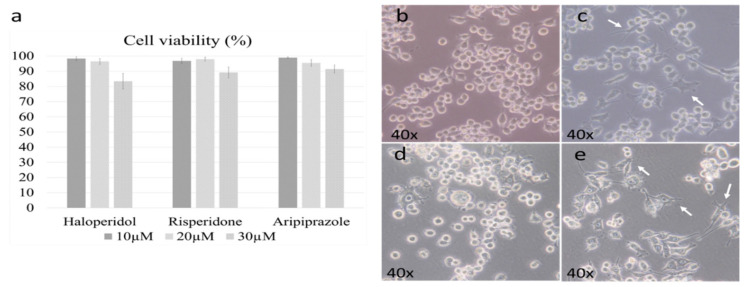
Cell viability and morphology after antipsychotic administration. Three different doses of antipsychotics were examined during the cell viability assay (10 µM, 20 µM, 30 µM). The dose of 20 µM was the highest one with satisfactory cell viability for all three antipsychotics (**a**). The morphology of control BV-2 microglial cells was mostly ameboid (**b**), with slight changes towards a more ramified state when treated with risperidone (**c**) and aripiprazole (**e**); representative cells are marked with white arrows. Cells treated with haloperidol retained a predominant ameboid morphology (**d**).

We first set out to determine the active and cytotoxic doses of each antipsychotic. The working doses were established according to previously published results by Kato et al. in the 6-3 microglial cell culture [28,29]. The cell viability threshold for experiments was set to 95%, with the optimal doses set due to their cytotoxicity. The chosen dosage was 20 µM for all antipsychotics, as the higher doses led to a marked reduction in viability (*p* < 0.005) (Figure 1a).

Previous research has shown that microglial morphology highly correlates with their function, with ameboid microglia being pro-inflammatory, while ramified ones are generally anti-inflammatory [34]. It should be noted that the degree of change is important, as their inflammatory activity can be considered as a spectrum that is followed by gradual changes in morphology [34]. Therefore, our first goal was to determine the effect of antipsychotics on the BV-2 cell morphology. The control cells were predominantly ameboid (Figure 1b), as we described in our previous research [33]. Administering risperidone (Figure 1c) and aripiprazole (Figure 1e) led to a slight ramification of cells, which was even more prevalent in aripiprazole. Haloperidol did not cause significant changes in cellular morphology, with most cells retaining an ameboid morphology (Figure 1d).

### 2.2. Effect of Antipsychotics on Inflammatory and Metabolic Phenotype Markers

We next set out to determine whether the change in morphology is followed by a change in the typical phenotype markers characteristic for BV-2 microglial cells. iNOS and Arg-1 are considered to be opposite enzymatic markers competing for arginine as a substrate, where the former signifies a metabolically active and pro-inflammatory phenotype, while the latter signifies an anti-inflammatory phenotype [35]. Further phenotype characterization was obtained using the classical and proven cell surface markers CD16/32 and CD86, which are pro-inflammatory phenotype markers, and CD206 as an anti-inflammatory phenotype marker [36].

**Figure 2:**
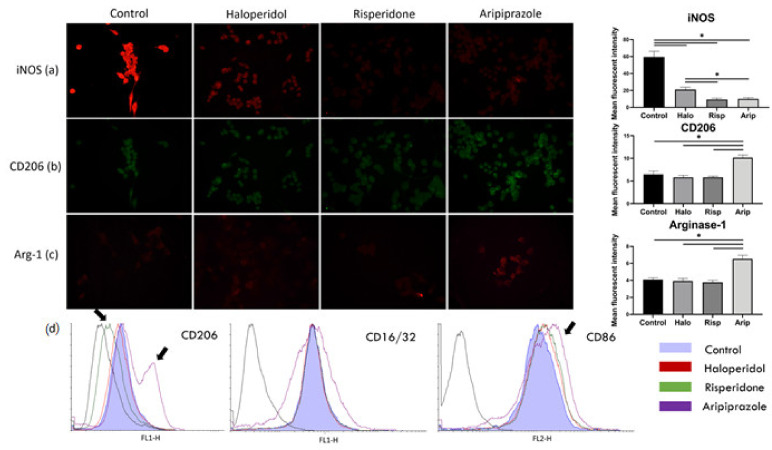
Effect of antipsychotics on inflammatory and metabolic phenotype markers. Expression of microglia polarization markers was assessed using immunofluorescence and flow cytometry. Control BV-2 microglial cells exhibited a high expression of iNOS, with low expressions of CD206 and Arg-1, constituting a pro-inflammatory phenotype (**a**). Administering antipsychotics led to a marked reduction in iNOS expression in all three tested substances (*p* < 0.0001), with most significant reductions upon administration of risperidone (*p* < 0.0001) and aripiprazole (*p* < 0.0001) (**a**). CD206 (**b**) and Arg-1 (**c**) expressions only increased when the cells were stimulated with aripiprazole (*p* < 0.0001). Similar was observed with flow cytometry (**d**), where aripiprazole administration led to an increase in cells expressing CD206 and CD86 (marked with black arrows). On the other hand, risperidone decreased the expression of CD206 in cells on flow cytometry (marked with black arrows). * Statistical significance was set at *p* ≤ 0.05 values.

The results of the immunofluorescence point to a significant reduction in iNOS mean fluorescent intensity after treatment with all three antipsychotics (F_3, 36_ = 380.0, *p* = 0.0015), with the highest statistically significant reduction in risperidone (*p* < 0.0001) and aripiprazole (*p* < 0.0001) treated cells (Figure 2a). The mean fluorescent intensity of CD206 and Arg-1 significantly increased after aripiprazole administration (*p* < 0.0001) compared to the control, haloperidol and risperidone samples (Figure 2b,c). On the other hand, there was no increase in the expression of mentioned markers in haloperidol (*p* = 0.622) and risperidone (*p* = 0.1205) samples compared to the control. Therefore, the BV-2 phenotype after treatment with haloperidol and risperidone was iNOS^low,^ CD206^low^ Arg-1^low^, while it was iNOS^low,^ CD206^high^ Arg-1^high^ in cells treated with aripiprazole (Figure 2a–c). All cells analyzed in flow cytometry had a predominant pro-inflammatory phenotype, with high CD86 and CD16/32 expression (Figure 2d). However, there was a significant number of cells with a higher CD206 and CD86 surface expression in aripiprazole-treated cells, while the risperidone-treated cells exhibited lower CD206 surface expression (Figure 2d). These results indicate that aripiprazole-treated cells display a distinct phenotype in both immunofluorescence and flow cytometry analysis towards a more anti-inflammatory phenotype, compared to the control, haloperidol and risperidone-treated cells (Figure 2).

### 2.3. Effect of Antipsychotics on the Cellular Metabolic Status

**Figure 3:**
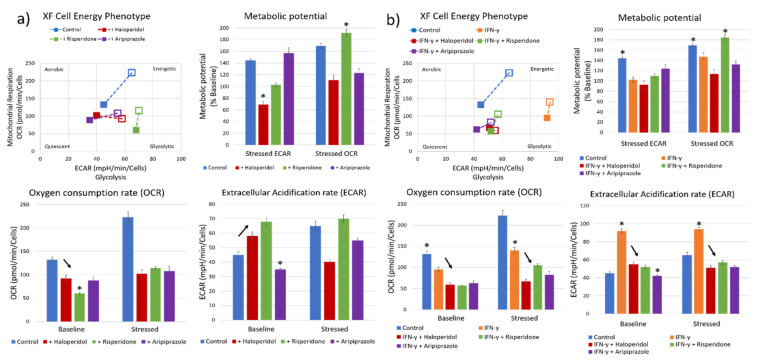
Effect of antipsychotics on the cellular metabolic status. Real-time metabolic status of BV-2 microglial cells was assessed using the Seahorse XF Analyzer. The metabolic phenotype of the cells changed significantly upon stimulation with antipsychotics (**a**), with a marked reduction in aerobic metabolism in all tested antipsychotics (F_3, 12_ = 158.6, *p* < 0.0001) and most significantly using risperidone (*p* < 0.0001). On the other hand, haloperidol (*p* < 0.05) and risperidone (*p* < 0.05) significantly increased the glycolytic metabolism compared to controls, while aripiprazole led to a reduction in glycolysis (*p* < 0.001), leading the cells to a more metabolically quiescent state. The greatest aerobic metabolic potential of cells under stress was present in control and risperidone-treated cells, while the glycolytic potential was highest in control and aripiprazole cells. Haloperidol-treated cells exhibited a reduced aerobic metabolic potential compared to baseline (*p* < 0.05). Cells pretreated with IFN-γ (**b**) exhibited a strong polarization towards glycolytic metabolism, which was successfully reversed by all three tested antipsychotics (F_4, 15_ = 721.6, *p* < 0.0001). Importantly, cells retained their metabolic potential, especially when treated with risperidone and aripiprazole. * Statistical significance was set at *p* ≤ 0.05 values.

Our next goal was to characterize the metabolic effects of antipsychotic administration, using a real-time cellular metabolic assay. We first measured the effects of antipsychotic administration on BV-2 microglial cells cultivated in 10% FCS DMEM medium. The control cells had a balanced metabolic state, with similar aerobic and glycolytic activity, and a sustained metabolic potential when stressed during the assay (Figure 3a). Cells pretreated with antipsychotics prior to the assay had a varied response to each substance, although with markedly reduced aerobic activity (oxygen consumption rate—OCR; mean values with standard deviation) with all three antipsychotics (F_3, 12_ = 158.6, *p* < 0.0001) (Figure 3a). Cells treated with risperidone (*p* < 0.05) and haloperidol (*p* < 0.05) exhibited significantly increased glycolytic metabolism (extracellular acidification rate—ECAR; mean values with standard deviation), while cells treated with aripiprazole had reduced glycolytic metabolism (*p* < 0.001) (Figure 3a). Stressing the cells revealed that the remaining metabolic potentials of cells vary between different substances. Aripiprazole-treated cells exhibited higher glycolytic metabolic reserves compared to the aerobic metabolism, with opposite results in cells treated with risperidone. Interestingly, haloperidol reduced the glycolytic metabolic potential, with an almost baseline aerobic potential (Figure 3a). These results indicate that each antipsychotic has different metabolic effects on the cells, although reducing aerobic metabolism is something that was common in all stimuli.

Recent research has shown that one of the possible changes that occur during acute psychosis is an upregulation of anaerobic glycolysis and increased lactate levels [37]. Therefore, we also pretreated the BV-2 microglial cells with a known microglial activator, IFN-γ, to induce a glycolytic metabolic state before administering antipsychotics. Cells that were treated with IFN-γ exhibited significantly higher baseline glycolytic activity and lower aerobic metabolic activity compared to the control cells (*p* < 0.0001) (Figure 3b). All three tested antipsychotics reduced both the aerobic and glycolytic metabolic activity, reversing the changes induced by IFN-γ (F_4, 15_ = 721.6, *p* < 0.0001; F_4, 15_ = 895.1, *p* < 0.0001) (Figure 3b). Aripiprazole reduced the glycolytic activity (*p* < 0.0001) to a higher degree than risperidone (*p* < 0.001) and haloperidol (*p* < 0.001), similar to what occurred in the cells without IFN-γ pretreatment. IFN-γ and risperidone-treated cells exhibited reduced glycolytic metabolic potential, while having significant aerobic metabolic reserves. On the other hand, cells treated with aripiprazole had a balanced metabolic potential (Figure 3b).

### 2.4. Effect of Antipsychotics on the mTORC1 Signaling Pathway

**Figure 4:**
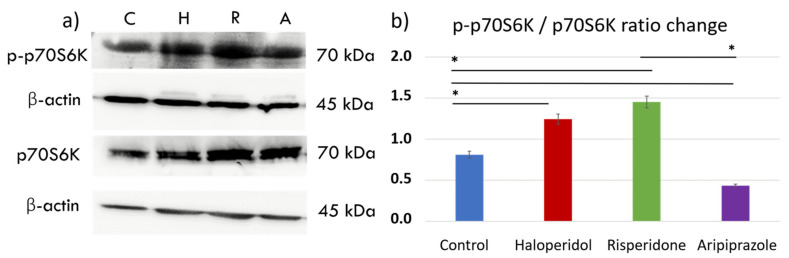
Effect of antipsychotics on the mTORC1 signaling pathway. Expression of the mTORC1 signaling pathway was assessed using Western blot. There were significant differences in p70S6K activity between control cells and those treated with antipsychotics (F_3, 12_ = 1.840, *p* < 0.0001). Control BV-2 microglial cells exhibited moderate activity of p70S6K (**a**,**b**), a downstream protein in the mTORC1 signaling pathway. Haloperidol (*p* < 0.0001) and risperidone (*p* < 0.0001) increased the expression of phosphorylated p70S6K (p-p70S6K), indicating mTORC1 activity. On the other hand, aripiprazole (*p* < 0.0001) significantly reduced the p-p70S6K/p70S6K ratio, indicating diminished mTORC1 activity compared to the control cells (**b**). * Statistical significance was set at *p* ≤ 0.05 values.

The mechanistic target of rapamycin (mTOR) pathway has been shown as a key pathway for microglial metabolic programming, with high pathway activity linked to increased glycolytic metabolism and pro-inflammatory action [38]. We sought to elucidate the effects of antipsychotic administration on the mTOR pathway by measuring downstream p70S6 kinase (p70S6K) signaling activity with Western blot (Figure 4a). There were significant differences in p70S6K activity between control cells and those treated with antipsychotics (F_3, 12_ = 1.840, *p* < 0.0001). Interestingly, antipsychotics haloperidol (*p* < 0.0001) and risperidone (*p* < 0.0001) increased the phosphorylated p70S6K (p-p70S6K) to p70S6K ratio, indicating greater mTORC1 activity compared to the control cells. (Figure 4b). On the other hand, aripiprazole reduced the activity of this pathway compared to the controls (*p* < 0.0001), indicating a divergent underlying mechanism of the tested antipsychotics in reducing the metabolic and inflammatory activity seen in previous experiments.

## 3. Discussion

In this research, we characterized the effects of antipsychotics haloperidol, risperidone and aripiprazole on BV-2 microglial cells in an in vitro setting, part of a field that is currently understudied. We have analyzed three characteristics of microglial cells that are interconnected with their function: morphology, phenotype polarization and cellular metabolism.

The morphology of microglial cells is integral to their function and can be considered as a direct representation of their activation status [39]. Changes in cellular ramification represent a gradual change towards an anti-inflammatory phenotype, as the degree of inflammatory activation correlates with morphology, with ameboid morphology being the most active [34]. In our research, antipsychotics induced slight changes in morphology, with aripiprazole and risperidone-treated cells displaying a more ramified morphology compared to those treated with haloperidol. A recent in vivo study by Bloomfield et al. demonstrated that haloperidol does not alter microglial morphology compared to the control [40], which is similar to what we observed in our setting. On the other hand, changes in microglial morphology towards ramification were described in a study by Kato et al., where murine 6-3 microglial cells attained a varied, multipolar shape after aripiprazole administration [41]. To the best of our knowledge, there are no studies that reported microglial morphological changes after the administration of risperidone, which induced slight ramification of BV-2 microglial cells in our study.

iNOS is a crucial metabolic marker necessary for murine microglia pro-inflammatory activity [35]. Progressive microglial activation and an increase in iNOS expression were present in an in vivo rat study using a neurodevelopmental model of schizophrenia, and importantly, the activation was successfully reversed using clozapine [42]. All three antipsychotics analyzed in our study reduced the expression of iNOS, although with differences as haloperidol did so to a lesser degree than risperidone and aripiprazole. Furthermore, aripiprazole increased the expression of CD206 and Arg-1, two anti-inflammatory phenotype markers, indicating a distinct phenotype. A reduction in iNOS expression was previously reported in 6-3 microglial cells stimulated by IFN-γ for risperidone and haloperidol, and the reduction was significantly higher in cells treated with risperidone, which is in line with our results as well [28]. Similar effects were seen by the same group when treating the cells with aripiprazole, although only with IFN-γ pretreatment [29]. Looking towards other antipsychotics, studies by Bian et al., reveal a similar effect in perospirone, ziprasidone and quetiapine on reducing the pro-inflammatory activity of microglial cells in an in vitro setting [27]. Our results, together with previous studies, indicate a trend that most antipsychotics can reduce the expression of proinflammatory markers in microglial cells, even though there are differences between substances. This is an important finding, as neuroinflammation is hypothesized to be an important aspect of schizophrenia pathophysiology [43].

Current research indicates that the energy demands of the brain in schizophrenia are increased, to the point that cells often shift to glycolytic pathways to meet energy demands [44]. The metabolic effect of antipsychotic treatment is a common topic of discussion and the cause of numerous side-effects, especially in second-generation antipsychotics such as risperidone or olanzapine [45]. There is a lack of studies covering the effects of antipsychotics of metabolic properties on microglial cells. Thus, we have performed a real-time metabolic assay of microglial cells treated with the three tested antipsychotics to assess their direct effect in modulating metabolic activities in the cells. All three antipsychotics lowered the aerobic metabolic rate; however, there were significant differences in the anaerobic metabolism. Haloperidol and risperidone increased the glycolytic activity of cells, in contrast to aripiprazole, which reduced it compared to control cells. Importantly, all antipsychotics were successful in ameliorating increased glycolytic metabolic activity in IFN-γ stimulated cells.

A proteome analysis of oligodendrocytes treated with antipsychotics revealed that haloperidol increased the proteins related to glycolysis, but risperidone did not [46], which is different from the functional result observed in our study. Opposite results for haloperidol were seen by Steiner et al., with an indirect indication of reduced glycolytic activity upon haloperidol stimulation in oligodendrocytes [47]. The predominant use of fat oxidation and a reduction in glycolysis in risperidone, olanzapine and clozapine were seen in an in vivo mice study by Klingerman et al., with no effect with aripiprazole [48]. We hypothesize that the divergence between our in vitro and the mentioned in vivo study could either be due to the difference of available nutrients in the cell medium or a cell-specific effect in microglia. Regarding aripiprazole, a study by Nagasaki et al. in PC12 cells showed that aripiprazole was superior to haloperidol in reducing oxidative stress due to adjustments in the glycolytic pathway, which is in line with our results [49]. Furthermore, aripiprazole has exhibited antioxidative properties via the inhibition of phorbol-myristate-acetate in microglial cells in vitro [41]. This is further demonstrated by studies of antipsychotic-induced oxidative stress in mice, as haloperidol induced oxidative stress that was not seen in aripiprazole [50]. Observing the clinical efficacy and profile, we can see that the metabolic syndrome is not as common in aripiprazole compared to risperidone or olanzapine, indirectly confirming these previous preclinical findings [51]. It is important to mention that these clinical findings have been called into question with a recent study and warrant further clinical research [52]. These studies generally indicate a different mechanism of metabolic activity in haloperidol, risperidone, and aripiprazole. Evidence of aripiprazole activity is the most consistent and points to a reduced metabolic activity with possible antioxidative potential in preclinical research, while the results are not as clear in cases of haloperidol and risperidone and require further investigation.

Our last goal was to try and elucidate a possible underlying mechanism for the effects of antipsychotic actions observed in earlier experiments. We tested the activity of the mTORC1 pathway as it is a key factor for metabolic reprogramming in microglial cells, in both glycolysis and oxidative phosphorylation [38]. Interestingly, the inhibition of mTOR activity suppressed glycolysis and the formation of reactive oxygen species when cells were stimulated with ATP and lipopolysaccharide (LPS), but caused the attenuation of aerobic metabolism only in ATP-stimulated microglial cells [38]. Inhibition of the mTORC1 pathway led to an increase in anti-inflammatory microglial activity following stroke in a study by Li et al. [53]. Cell proliferation is another function attributed to mTOR activity, while cells with strong mTOR inhibition have reduced viability [54]. Finally, mTORC1-dependent signaling has been linked in post-mortem animal studies with schizophrenia, specifically the hypofunction of the ribosomal protein S6 [55].

We used p70S6K to analyze mTORC1 activity, as the phosphorylation of p70S6K is strongly indicative of mTORC1 activity [56]. All three antipsychotics significantly changed the p-p70S6K / p70S6K ratio, albeit in a different way. Haloperidol and risperidone increased the ratio, indicating increased mTORC1 activity compared to control BV-2 microglial cells. Aripiprazole, on the other hand, significantly decreased the ratio and phosphorylation of p70S6K, leading to reduced mTORC1 activity in treated cells. These results are in line with observations from the real-time metabolic assays, although all antipsychotics lowered the expression of iNOS, indicating a different mechanism for metabolic reprogramming between the tested antipsychotics. Recent studies have shown that haloperidol has capabilities in increasing mTORC1 activity in neurons, which was also linked to extrapyramidal motor side effects [57,58,59]. Increased mTORC1 activity and glycolysis was also seen in olanzapine [60], a second-generation antipsychotic, similar to what we found in cells treated with risperidone. In contrast to our results, Ibarra-Lecue et al. have shown that only haloperidol, and not risperidone, increased the activation of the AKT/mTORC1 pathway [55]. However, a drug-regulated transcriptional study by Korostynski et al. revealed that second-generation antipsychotics, including risperidone, strongly induced the transcription of mTOR pathway-related genes [61]. mTOR is a master regulator of cellular growth and metabolic activity, and its hyperactivation has been linked to obesity and type 2 diabetes [62,63]. The increased activity that we observed with haloperidol, and especially risperidone, could be one of the mechanisms of metabolic side-effect development that can be seen in long-term use. Clinically, risperidone has higher effects on the development of metabolic syndrome and weight gain than haloperidol, even though it has also been shown to impact the blood levels of triglycerides and cholesterol [64]. Interestingly, increased mTORC1 activity can lead to an increase in triglyceride secretion from the liver [65].

On the other hand, aripiprazole induced lower mTORC1 activity in our cells, which could have a significant impact and a possible mechanism of both its anti-inflammatory and antioxidant action. An in vitro study by Sato-Kasai et al. demonstrated that aripiprazole inhibits polyl:C-induced microglial activation possibly via the transient receptor potential in melastatin 7 (TRPM7) [30]. TRPM7 and the mTORC1 pathway have been linked to the function of TRPM7 kinase in facilitating cellular proliferation and growth. Interestingly, mTORC1 activation is significantly reduced in TRPM7-deficient cells [66], which could be a possible mechanism by which aripiprazole reduces the mTORC1 pathway activity. Furthermore, aripiprazole was the only antipsychotic that induced AMP-activated protein kinase (AMPK) phosphorylation in PC12 cells in an in vitro comparative study [67]. AMPK and mTOR signaling pathways can be seen as functional opposites, but with significant cross talk in numerous cell types [68]. Activation of the AMPK signaling pathway in microglia is followed by anti-inflammatory responses and polarization [69], which we only observed with aripiprazole. Thus, blocking mTORC1 activity via TRPM7 could be the reason why aripiprazole-treated cells exhibited less glycolytic activity and increased expression of anti-inflammatory markers when compared to haloperidol and risperidone. Lower mTOR activity is linked with beneficial effects on aging and cellular health [70]. However, long-term aripiprazole use can also induce metabolic side-effects in patients, albeit to a significantly lesser extent when compared to the second-generation antipsychotics [64]. The question remains whether blocking mTOR function could be the mechanism by which these side-effects occur, or if it is a consequence of changes in other signaling pathways that still need to be characterized.

The limitations of our study are in-vitro conditions and the use of immortalized BV-2 microglial cells; therefore, additional studies on primary cultures and in vivo settings are required to confirm the results we obtained.

## 4. Materials and Methods

### 4.1. Cells

BV-2 microglial cell line, which exhibited the phenotypical and functional properties of reactive microglial cells [31], was grown in Dulbecco’s Modified Eagle’s medium (DMEM) (PAN Biotech, Aidenbach, DE) and supplemented with 10% (*v*/*v*) fetal calf serum (FCS), 2mM L-glutamine, 100 mg streptomycin and 100 U penicillin (GIBCO, Gran Island, NY, USA). The cells were cultured in Petri dishes at 37 °C with 5% CO_2_ and saturated humidity. The culture conditions of the BV-2 cell line and the effects of various FCS supplements were described in our previous study, and we chose cultivation with the addition of FCS in order to keep the microglial cells in an active state [33].

### 4.2. In Vitro Cell Viability Assay

Cell viability was assessed using the Countess FL Automated Cell Counter (Thermo Fisher Scientific, Waltham, MA, USA), an automated technique which has been validated for assessing cell viability [71]. The viability was assessed before each experiment as part of standard protocol and cell counting. We proceeded with the experiment if the cell viability was ≥95% in both cultivation conditions.

### 4.3. Reagents and Antibodies

Haloperidol, risperidone, and aripiprazole were acquired from Sigma Aldrich (Sigma Aldrich, St. Louis, MO, USA) and dissolved in DMSO per manufacturer’s instructions. iNOS (Thermo Fisher Scientific, Waltham, MA, USA) and CD206 (BioRad, Hercules, CA, USA) monoclonal antibodies were used for immunofluorescence. Secondary antibodies used were Alexa 488 goat α-mouse IgG_1_, Alexa 488 and 555 goat α-rat IgG_2a_ and Alexa 555 goat α-rabbit secondary reagents (Thermo Fisher Scientific, Waltham, MA, USA). The antibodies used in flow cytometry analysis were anti-CD86-PE (BD Biosciences, San Jose, CA, USA, SAD) and anti-CD16/32-FITC (BD Biosciences, San Jose, CA, USA, SAD). mTOR signaling was assessed with anti-phospho-p70S6K and anti-p70S6K antibodies (Cell Signaling Technologies, Danvers, MA, USA), while anti β-Actin (Sigma Aldrich, St. Louis, MO, USA) was used as a loading control. Secondary antibody for Western blot analysis was HRP conjugated goat α-rabbit (Cell Signaling Technologies, Danvers, MA, USA). Real-time metabolism assay was performed using IFN-γ (Genentech, San Francisco, CA, USA), LPS (Sigma Aldrich, St. Louis, MO, USA) and FCCP/Oligomycin as part of the Cell Energy Phenotype Test Kit (Agilent, Santa Clara, CA, USA).

### 4.4. Immunofluorescence

Cells were analyzed 24 h after antipsychotic administration. Cells grown in monolayers on glass coverslips in twenty-four well tissue-culture plates were rinsed with phosphate buffered saline (PBS), fixed with 4% paraformaldehyde (PFA) for 20 min, permeabilized with 0.1% Triton X-100 for 10 min and blocked with 2% fetal calf serum in PBS for 30 min. Labeling was performed using primary and coupled secondary antibodies for 1 h at room temperature. Images were acquired using the fluorescent microscope Olympus BX51 (magnification of 400–1000×). All immunofluorescence experiments were repeated a minimum of three times, with a minimum of 10 images from all experiments. The images were analyzed with the Zen Blue software for obtaining the mean fluorescent intensity.

### 4.5. Flow Cytometry

Cells were cultured in 12-well tissue culture plates and incubated at 37 °C and 5% CO_2_. Twenty-four hours after antipsychotic administration, cells were trypsinized, neutralized and cellular suspension was centrifugated for 5 min at 1500 rpm. After the centrifugation, cells were resuspended and divided into flow cytometry tubes, with approximately 10^5^ cells representing one sample. Subsequently, primary followed by corresponding secondary antibody was added to each sample. Between the incubation steps, samples were washed with FACS buffer and centrifugated for 2 min at 2000 rpm. Samples were analyzed on FACSCalibur flow cytometry (BD Biosciences, Franklin Lakes, NY, USA) using The CellQuest Pro (BDBiosciences, Franklin Lakes, NY, USA) program. The results were processed with Flowing Software (Turku, Estonia) application.

### 4.6. Western Blot Analysis

Cellular extracts were prepared in RIPA lysis buffer supplemented with protease inhibitor and/or phosphatase inhibitor cocktail set (Roche, Basel, CH). Proteins were separated by sodium dodecyl sulphate polyacrylamide gel electrophoresis (SDS-PAGE) and blotted onto a PVDF Western blotting membrane (GE Healthcare Limited, Amersham, UK). Membranes were washed in Tris/HCl-buffered saline (TBS), blocked with 5% non-fat milk for 1 h at room temperature, and incubated with primary antibodies at 4°C overnight. After 1 h incubation at room temperature with horseradish peroxidase-coupled secondary antibodies, signals were detected with enhanced chemiluminescence Amersham ECL Prime Western Blotting Detection Reagent (GE Healthcare Limited, Amersham, UK) according to standard methods. Western blot quantification was performed using the Image studio software (LI-COR Biosciences, Lincoln, NE, USA) over three experiment samples.

### 4.7. Real-Time Metabolism Assay

ECAR and OCR measurements were made with an XF-24 Extracellular Flux Analyzer (Agilent Santa Clara, CA, USA), proven in this cell line [72]. A utility plate containing calibrant solution (1 mL/well) together with the plates containing the injector ports and probes were placed in a CO_2_-free incubator at 37 °C overnight. The following day, cells were plated at 0.2 × 10^6^ cells/well of a 24-well Seahorse plate (same-day seeding) with one well per row of the culture plate containing only supplemented media without cells, as a negative control. Cell viability was assessed, as described previously, prior to seeding. Before the assay, media were removed from cells and replaced with glucose, pyruvate and glutamine-supplemented XF assay buffer (500 mL/well) and the cell culture plate was placed in a CO2-free incubator for at least 1 hr. Antipsychotics were administered prior to the assay, while IFN-γ stimulation was performed 24 h prior to the cell seeding on a Seahorse plate. Inhibitors (Oligomycin and carbonyl cyanide-4-(trifluorome-thoxy)phenylhydrazone (FCCP)) were added to the appropriate port of the injector plate. This plate together with the utility plate was run on the Seahorse for calibration. Once complete, the utility plate was replaced with the cell culture plate and run on the Seahorse XF-24. All cultivation conditions were conducted in quadruplicates to obtain the mean ECAR and OCR values provided in the results.

### 4.8. Statistical Analysis

Statistical analysis was performed using Statistica v13 program (TIBCO Software, Palo Alto, CA, USA). The Graph-Pad Prism program (GraphPad Software Inc., San Diego, CA, USA) and Microsoft Excel (Microsoft corp., Redmond, WA, USA) were used for graphic presentation. Normal distribution was assessed using the Kolmogorov–Smirnov test, and the Shapiro–Wilk test in case of a small number of samples. Student’s t test was used to compare two independent samples. One-way ANOVA and the Tukey’s post hoc test were used between three or more independent samples. Signals of fluorescent dots were quantified for each cell by Zen Blue imaging software (Zeiss, Oberkochen, DE), with 10 images analyzed over three experiments per condition. Immunofluorescence results were reported as mean fluorescent intensity with standard deviations. Real-time metabolic analysis assay results are presented as mean values with standard deviations of ECAR and OCR values. Western blot quantification was performed in Image studio software by LI-COR Biosciences, considering three independent experiment samples. Statistical significance was set at *p* ≤ 0.05 values.

## 5. Conclusions

In this study, we demonstrate the varying effects of haloperidol, risperidone and aripiprazole administration in BV-2 microglial cells. All three tested antipsychotics were successful in reducing the pro-inflammatory action of microglial cells, although only aripiprazole increased the expression of anti-inflammatory markers. The most significant differences in the possible mechanisms of action are seen in the real-time metabolic assays and changes in mTORC1 signaling pathway activity, where our results shed some new light on the effects of haloperidol, risperidone and aripiprazole action in microglial cells. Importantly, all tested antipsychotics were effective in curbing the IFN-γ-induced activation in BV-2 microglial cells.

Based on our results and the current literature, we propose that aripiprazole-induced mTORC1 inhibition is an important mechanism of action in microglial cells that leads to an anti-inflammatory shift and balanced metabolic action. On the other hand, the upregulation of mTORC1 activity by risperidone could be connected to the metabolic side-effects that are observed in long-term treatment. Similar results can be seen in haloperidol, albeit to a lesser extent. Further studies are required to elucidate the possible mechanisms of haloperidol and risperidone anti-inflammatory action, which do not follow the same pattern as aripiprazole.

## Data Availability

The data presented in this study are available on request from the corresponding author.
